# Clinical Characteristics of 5 COVID-19 Cases With Non-respiratory Symptoms as the First Manifestation in Children

**DOI:** 10.3389/fped.2020.00258

**Published:** 2020-05-12

**Authors:** Xiaofang Cai, Yaoling Ma, Songbo Li, Yan Chen, Zhihui Rong, Wenbin Li

**Affiliations:** ^1^Emergency Department, Wuhan Children's Hospital (Wuhan Maternal and Child Healthcare Hospital), Tongji Medical College, Huazhong University of Science and Technology, Wuhan, China; ^2^Department of Pediatrics, Union Hospital, Tongji Medical College, Huazhong University of Science and Technology, Wuhan, China; ^3^Department of Pediatrics, Tongji Hospital, Tongji Medical College, Huazhong University of Science and Technology, Wuhan, China

**Keywords:** novel coronavirus disease 2019, pediatrics, clinical characteristics, first manifestation, non-respiratory symptoms

## Abstract

An outbreak of the novel coronavirus disease 2019 (COVID-19) occurred in Wuhan, China, in December 2019, which then rapidly spread to more than 80 countries. However, detailed information on the characteristics of COVID-19 in children is still scarce. Five patients with non-respiratory symptoms as the first manifestation were hospitalized from the emergency department, and were later confirmed to have COVID-19, between 23 January and 20 February 2020, at the Wuhan Children's Hospital. SARS-CoV-2 nucleic acid detection was positive for all the patients. Four of the patients were male and one was female, and their ages ranged from 2-months to 5.6 years. All lived in Wuhan. One patient had a clear history of exposure to SARS-CoV-2, one had a suspected history of exposure, while the others had no exposure history. For three of the five patients, the primary onset disease required an emergency operation or treatment, and included intussusception, acute suppurative appendicitis perforation with local peritonitis, and traumatic subdural hemorrhage with convulsion, while for the other two it was acute gastroenteritis (including one patient with hydronephrosis and a stone in his left kidney). During the course of the disease, four of the five patients had a fever, whereas one case had no fever or cough. Two patients had leukopenia, and one also had lymphopenia. In the two cases of severe COVID-19, the levels of CRP, PCT, serum ferritin, IL-6, and IL-10 were significantly increased, whereas the numbers of CD3+, CD4+, CD8+ T lymphocytes, and CD16 + CD56 natural killer cells were decreased. We also found impaired liver, kidney, and myocardial functions; the presence of hypoproteinemia, hyponatremia, and hypocalcemia; and, in one case, abnormal coagulation function. Except for one patient who had a rotavirus infection, all patients tested negative for common pathogens, including the influenza virus, parainfluenza virus, respiratory syncytial virus, adenovirus, enterovirus, mycoplasma, Chlamydia, and Legionella. Chest CT images of all the patients showed patches or ground-glass opacities in the lung periphery or near the pleura, even large consolidations. This case series is the first report to describe the clinical features of COVID-19 with non-respiratory symptoms as the first manifestation in children.

## Introduction

A novel coronavirus was identified in December 2019 in Wuhan, Hubei Province, China, following a series of pneumonia cases of unknown etiology in people with a history of exposure to the Huanan seafood market (1, 2). The World Health Organization (WHO) announced that the outbreak constituted a public health emergency of international concern and named the disease COVID-19, short for “coronavirus disease 2019” ^1^. Soon after, the virus was officially named as severe acute respiratory syndrome coronavirus 2 (SARS-CoV-2) by the International Committee on Taxonomy of Viruses (ICTV). As of 10:00 AM on 5 March 2020, SARS-CoV-2 had spread to 85 countries^1^. The age of infected people range from 1.5 days to 96 years, and there is no significant difference in susceptibility between genders. The groups at high-risk of critical illness and death are the elderly with underlying conditions such as diabetes, hypertension, and cardiovascular disease, or those over the age of 60. With the outbreak reaching its peak, there has been a gradual increase in the number of reported cases of infected children, including neonates (3, 4).

Similar to that observed for severe acute respiratory syndrome (SARS) and Middle East respiratory syndrome (MERS), COVID-19 in children has low morbidity, and most either develop mild symptoms or are asymptomatic. The few severe cases have usually exhibited underlying or coexisting medical conditions (3–5). Indeed, some of the children with confirmed COVID-19 did not show respiratory symptoms as the first manifestation. They had a concealed onset, and it was only after chest computerized tomography (CT) scans for other diseases that the signs of viral pneumonia were found, with the diagnosis of SARS-CoV-2 infection being subsequently confirmed by nucleic acid detection. Five patients diagnosed with COVID-19 and not presenting with respiratory symptoms as the first manifestation were admitted to the hospital from the emergency department between 23 January and 20 February 2020. Attention was focused on how to reduce potential transmission and cross infection, and how to identify, isolate, and treat them at an early stage. We analyzed the clinical features associated with these five cases. This case series was approved by the institutional ethics board of Wuhan Children's Hospital, affiliated to Tongji Medical College of Huazhong University of Science and Technology (WHCH 2020007). Each child's legal guardian provided written informed consent for participation in this study.

In this report, the clinical stage of the patients was classified according to “diagnosis, treatment, and prevention of 2019 novel coronavirus infection in children: experts' consensus statement” (6). Severe COVID-19 was defined when the pediatric patients met any of the following criteria: (1) increased respiratory rate: ≥70 times/min (<1 year), ≥50 times/min (≥1 year) (after ruling out the effects of fever and crying); (2) oxygen saturation <92%; (3) hypoxia: assisted breathing (moans, nasal flaring, and three concave sign), cyanosis, intermittent apnea; (4) disturbance of consciousness: somnolence, coma, or convulsion; (5) food refusal or feeding difficulty, with signs of dehydration. Critically ill COVID-19 was defined when the pediatric patients met any of the following criteria and required ICU care: (1) respiratory failure which requires mechanical ventilation; (2) shock, and (3) combined with other organs failure.

## Cases

### Case 1

**Figure 1:**
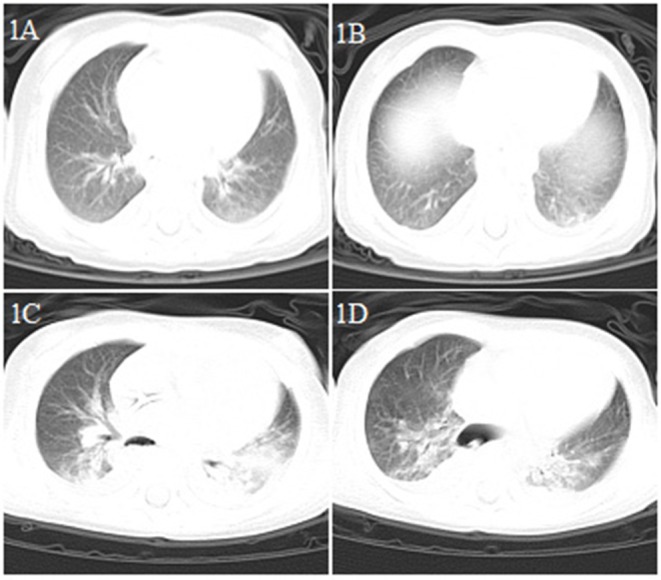
Chest CT images for case 1 showing a small dense shadow in the left lung on day 4 after symptom onset **(A,B)**; bilateral ground-glass opacity, large consolidation, and bilateral pleural effusion on day 9 after symptom onset **(C,D)**.

A 10-month-old female infant was admitted to the hospital from the emergency department with suspicion of intussusception, presenting with paroxysmal crying and restlessness, vomiting, and currant jelly-like stool for 30 h (Table 1). Fever was detected soon after arrival at the emergency department (body temperature 38.6°C), but no respiratory symptoms were observed. The patient had no history of exposure to COVID-19, and successfully underwent diagnostic air enema soon after admission. However, the child subsequently developed apathy, drowsiness, and a high fever (the maximum body temperature was 39.8°C). Owing to intermittent convulsion, the child was immediately transferred to the pediatric intensive care unit (PICU). Blood counts showed leukopenia (3.27 × 10^9^/L), lymphopenia (1.06 × 10^9^/L), and thrombocytopenia (29 × 10^9^/L), and increased levels of C-reactive protein (CRP) (up to 202 mg/L) and procalcitonin (PCT) (>100 ng/ml). The patient also exhibited multiple organ dysfunction (liver, kidney, myocardium); prolonged prothrombin time (PT, 22.1 s) and activated partial thromboplastin time (APTT, 76 s); increased levels of D-dimer (40.34 mg/L); significantly increased concentrations of serum ferritin (1179.11 ng/ml), interleukin (IL)-6 (3868.86 pg/ml), and IL-10 (326.93 pg/ml); low albumin; hyponatremia and hypocalcemia; and reduced numbers of CD3+, CD4+, CD8+ T lymphocytes, and CD16 + CD56 natural killer (NK) cells (Table 2). The ambulatory electroencephalogram (EEG) background was diffuse low amplitude slow wave combined with considerable fast wave activity. On the day of admission, a chest CT scan showed a small dense shadow in the left lung (Figures 1A,B); 5 days later, a reexamination showed that the lung lesions had deteriorated, and patchy ground-glass opacities and large scale consolidation were seen in both lungs with bilateral pleural effusion (Figures 1C,D). The patient tested positive twice for SARS-CoV-2 nucleic acid in throat swab samples. On day 9 after admission, the patient had obvious abdominal distention, dark-red bloody stools, and coffee dreg-like gastric contents could be seen during continuous gastrointestinal decompression. Doppler ultrasonography of the peritoneum suggested a large amount of abdominal dropsy. After intubation and exploratory laparotomy, necrosis of the proximal ileus of the small intestine was found, and resection was performed. The patient received blood purification (plasma exchange, continuous renal replacement therapy [CRRT]), antibiotics (meropenem, linezolid), antivirals (ribavirin intravenous drip, interferon alpha-2b atomization), dopamine and dobutamine to improve circulation, a methylprednisolone intravenous drip and gamma globulin infusion in PICU, and died due to multiple organ failure.

**Table 1:** Epidemiology and clinical characteristics of five COVID-19 cases.

	**Case 1**	**Case 2**	**Case 3**	**Case 4**	**Case 5**
**Gender (M/F)**	**F**	**M**	**M**	**M**	**M**
Age (y/m)	10 m	5.6 y	8 m	1.2 y	2 m
Exposure history	No	Grandmother confirmed	No	No	Parents and grandparents suspected
First clinical manifestation	Paroxysmal crying, vomiting, currant jelly-like stool	Abdominal pain	Convulsion	Vomiting, diarrhea	Drowsiness and poor feeding, diarrhea
Time from illness onset to fever	30 h	2 days	6 h	5 days	—
Fever at presentation or prior to presentation	At presentation	No	At presentation	At presentation	No
Highest temperature (°C) during the course	39.8	39	38.5	37.9	—
Days from illness onset to admission	1.25	2	0.25	6	3
Underlying or coexisting disease	Intussusception	Acute suppurative appendicitis perforation with localized peritonitis	Traumatic brain injury	Hydronephrosis and stone in the left kidney, rotavirus infection	No
Complications	Shock; MODS (liver, kidney, myocardium, blood coagulation, intestinal); acute respiratory failure	No	No	Acute respiratory failure; acute heart failure; shock; MODS (liver, kidney, myocardium)	Myocardium function damaged
**Treatment**					
Antiviral therapy	Intravenous ribavirin, aerosol inhalation of interferon	Aerosol inhalation of interferon	Intravenous ribavirin, aerosol inhalation of interferon	Intravenous ribavirin, aerosol inhalation of interferon	Aerosol inhalation of interferon
Methylprednisolone	2 mg/(kg·d) × 5d	No	No	2 mg/(kg·d) × 3d	No
Intravenous immunoglobulin	500 mg/(kg·d) × 3d	No	No	500 mg/(kg·d) × 4d	No
Oxygen inhalation	Nasal cannula	No	Nasal cannula	Nasal cannula	No
Invasive mechanical ventilation	Yes	No	No	Yes	No
Blood purification	PE+CRRT	No	No	PE+CRRT	No
Length of stay (days)	36	10	14	17	15
Outcomes	Died	Cured	Cured	Cured	Cured

**Table 2:** Laboratory findings of the five cases.

	**Normal range**	**Case 1**	**Case 2**	**Case 3**	**Case 4**	**Case 5**
White blood cell count, × 10^9^/L	5.5–12	3.27	3.79	18.16	11.96	7.36
Neutrophil count, × 10^9^/L	1.08–5.9	2.08	1.2	8.14	7.77	1.86
Lymphocyte count, × 10^9^/L	1.15–6	1.06	2.01	8.93	2.48	4.2
Platelet count, × 10^9^/L	100–378	29	247	386	184	338
Urine analysis		PRO3+, BLD3+	Normal	Normal	PRO±, BLD3+	—
Stool routine		Positive occult blood	Normal	Normal	Positive occult blood	Loose stool
CRP, mg/L	0–3	202	5.01	24.8	69.1	5
PCT, ng/mL	≤ 0.05	>100	0.46	0.09	2.66	—
Serum ferritin, ng/mL	12–135	1179.11	—	30.3	1796.8	—
**Cytokine, pg/mL**						
IL-2	0–11.4	1.75	—	2.00	1.32	1.43
IL-4	0–12.9	3.01	—	3.78	3.37	0.86
IL-6	0–20.9	3868.86	—	70.83	177.86	7.06
IL-10	0–5.9	326.93	—	6.68	26.85	8.45
TNF-α	0–5.5	2.15	—	2.08	3.52	1.01
IFN-r	0–17.3	16.98	—	3.74	2.26	8.35
Albumin, g/L	35–50	20.9	46.7	41.2	31.8	39.1
Alanine aminotransferase, U/L	9–52	375	20	50	54	21
Aspartate aminotransferase, U/L	15–46	1,093	31	50	124	46
Blood urea nitrogen, mmol/L	2.5–6.1	22.84	5.94	—	15.91	2.00
Creatinine, μmol/L	46–92	206.3	24.5	—	224.5	17.7
**Electrolyte, mmol/L**						
K^+^	3.5–5.1	3.49	4.05	4.09	5.46	4.59
Na^+^	137–145	133.6	138.2	136.6	129.2	136.9
Cl^−^	98–107	104	100.1	98.7	94.7	97.3
Ca^2+^	2.1–2.55	1.32	2.39	2.27	1.87	2.55
Lactate dehydrogenase, U/L	161–371	3,171	242	363	751	562
Creatine kinase, U/L	30–170	20,702	84	107	177	97
Creatine kinase—MB, U/L	0–24	840	14	41	98	90
Troponin (ng/mL)	0–0.014	0.007	—	0.01	0.272	—
NT-proBNP (pg/mL)	0–300	>9,000	—	395	>9,000	—
PT, s	10.2–13.4	22.1	11	11.2	14.3	9.6
APTT, s	25.7–39	76	30.3	31	31.8	47.7
Fibrinogen, g/L	2–4	3.08	2.1	2.89	2.47	2.45
D-dimer, mg/L	0–0.55	40.34	—	—	—	1.18
**TBNK lymphocyte detection, per/μL**						
CD3+T	805–4459	268	—	5,643	705	—
CD8+T	314–2080	102	—	1,869	318	—
CD4+T	345–2350	155	—	3,110	342	—
CD16+CD56 NK	210–1514	11	—	1,695	6	—
CD19+B	240–1317	820	—	9.21	1,180	—
CD4+/CD8+T (Th/Ts)	0.96–2.05	1.52	—	1.66	1.07	—
Common pathogens		Neg	Neg	Neg	Rotavirus	Neg

*CRP, C-reactive protein; PCT, procalcitonin; PT, prothrombin time; APTT, activated partial thromboplastin time; IL, interleukin; NK, natural killer*.

### Case 2

**Figure 2:**
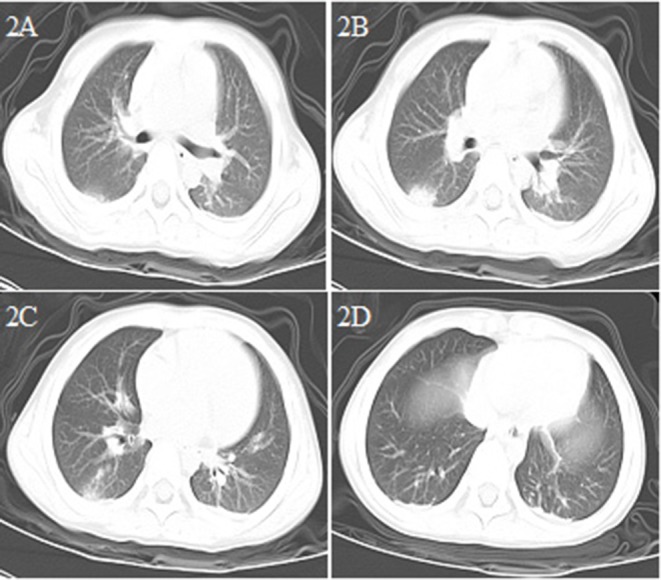
Chest CT images for case 2 showing a round-like mass shadow in the dorsal segment of the lower right lobe with a slight ground-glass opacity on the edge, a strip shadow in the local area of the left lower lobe, and an arc dense under the bilateral pleura on day 3 after symptom onset **(A–D)**.

A 5.6 year-old boy was admitted to the hospital for acute suppurative appendicitis perforation with localized peritonitis accompanied by continuous lower right abdominal pain for 2 days (Table 1). After admission, emergency surgery was immediately performed. Fever was detected before preparing for the operation (body temperature 38°C). Although the patient showed no respiratory symptoms or signs of COVID-19 and a preoperative chest X-ray was normal, a chest CT scan was performed nonetheless the day after the operation given that the patient had close contact with his grandmother who was a COVID-19 patient; the scan showed the presence of viral pneumonia (Figures 2A–D). The patient tested positive twice for SARS-CoV-2 nucleic acid in throat swab samples. The patient subsequently received antibiotics (cefoperazone sodium/sulbactam sodium), aerosol inhalation of interferon α-2b, and other supportive treatment. He was hospitalized for 10 days and then recovered.

### Case 3

**Figure 3:**
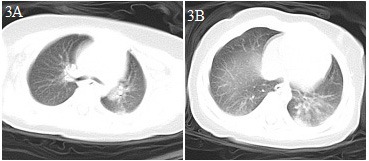
Chest CT images for case 3 showing a patchy dense shadow in the posterior part of the left lung with a blurred boundary on day 2 after symptom onset **(A,B)**.

An 8-month-old male infant came to the emergency department because of a 6 h episode of intermittent convulsion (Table 1). Considering that the patient had suffered a head trauma 3 days before, an emergency head CT scan was immediately performed, and a right frontal subdural hemorrhage was found. At the same time, he developed a fever (body temperature 38.5°C). Although the patient had no contact history with a COVID-19 patient and no respiratory symptoms, he nevertheless underwent a chest CT scan before admission to the PICU. The CT scan image showed a patchy dense shadow with a blurred boundary in the posterior part of the left lung (Figures 3A,B). The patient was admitted to the hospital with right frontal traumatic subdural hemorrhage and suspected COVID-19. He subsequently tested positive for SARS-CoV-2 nucleic acid. The patient received hemostasis, an anticonvulsant, interferon α-2b aerosol inhalation, and ribavirin intravenous drip treatments, and was cured. He was discharged from the hospital after a 14 day stay.

### Case 4

**Figure 4:**
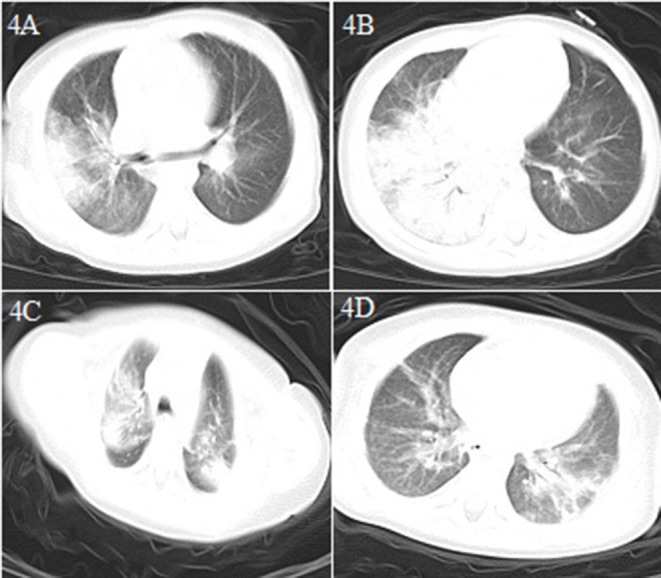
Chest CT images for case 4 showing a large dense shadow with some consolidation in the right lung and a few high-density shadows in the upper lobe of the left lung on day 6 after symptom onset **(A,B)** and patchy dense shadows mixed with ground-glass opacity in the bilateral upper lobe tip, right middle lobe, and left lower lobe, and air bronchogram inside on day 15 after symptom onset **(C,D)**.

A 1.2 year-old boy was transferred directly from the emergency department to the PICU because of intermittent diarrhea, vomiting for 6 days, and fever, lethargy, and shortness of breath for half a day in the emergency observation room (Table 1). He had no other respiratory symptoms such as a runny nose and cough, and no definite COVID-19-exposure history. He had a history of hydronephrosis and a stone in the left kidney. The initial etiology detection indicated rotavirus infection. Leukocyte and lymphocyte counts were normal, the CRP level was increased (69.1 mg/L), and the patient showed dysfunction of the liver, kidney, and myocardium. Blood coagulation was normal; serum ferritin (1796.8 ng/ml) levels were increased; the numbers of CD3+ and CD4+ T lymphocytes and CD16 + CD56 NK cells were decreased; and the levels of IL-6 (177.86 pg/ml) and IL-10 (26.85 pg/ml) were significantly increased (Table 2). A chest CT scan showed large consolidation in the right lung and a few high-density shadows in the upper left lung (Figures 4A–D). Nucleic acid testing for SARS-CoV-2 was negative on the second and seventh days and positive on the eighth day after admission. Blood oxygen saturation decreased rapidly soon after admission, with only 75–80% of the blood oxygen saturation being maintained after regular oxygen inhalation. Intubation and mechanical ventilation were immediately performed. As his condition continued to worsen and he had no urine, blood purification (plasma exchange, CRRT) was performed. At the same time, he was treated with vasoactive substances (dopamine, milrinone) to improve circulation, a cardiotonic (cediland), antibiotics (meropenem, linezolid), antiviral drugs (ribavirin intravenous drip and interferon α-2b atomization), a methylprednisolone intravenous drip, and other supportive treatments (gamma globulin, plasma albumin) and the patient was cured and discharged after 17 days of hospitalization. This was the first severe case of child COVID-19 in China (4).

### Case 5

**Figure 5:**
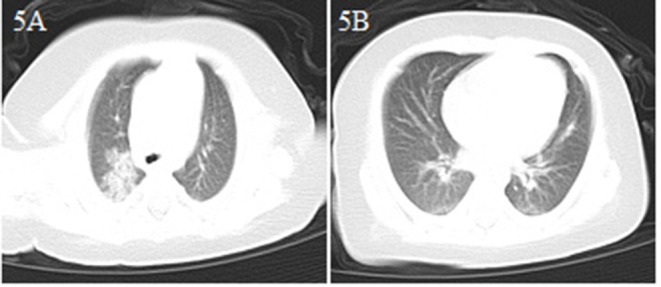
Chest CT images for case 5 showing bilateral scattered spots of shadows and consolidation in the upper right lobe on day 3 after symptom onset **(A,B)**.

A 2-month-old male infant came to the emergency department because of drowsiness and poor feeding for 3 days, and diarrhea for 2 days (Table 1). Although the patient had no fever or cough, because of a suspicion of contact history with COVID-19 patients (both parents and grandparents were suspected of having COVID-19), a chest CT scan was performed and showed bilateral lung pneumonia with local consolidation (Figures 5A,B). The patient was admitted to the hospital with suspected COVID-19. He had no fever and no respiratory symptoms, and his vital signs were stable. The patient received interferonα-2b atomization and was cured. He was discharged from the hospital after a 15 day stay (Table 2).

## Discussion

Wuhan Children's Hospital was the first to be assigned, by the government, for the treatment of children with COVID-19 in Hubei Province, China. During the outbreak, only the fever clinic and emergency department were open for outpatients. All outpatients required pre-examination and a triage system was employed. All medical staff in the emergency department took the required level 2 protective measures. If emergency endotracheal intubation or sputum suction was necessary, level 3 protective measures were to be taken.

The five cases were interesting because the patients initially had no respiratory symptoms at presentation; instead, they had to seek medical advice for unrelated problems in the emergency department. Three of the cases (cases 1, 2, and 3) had to be admitted to the hospital for an emergency operation or treatment. Cases 4 and 5 mainly showed gastrointestinal symptoms at the onset of illness. Case 4 was transferred directly from the emergency department to the PICU in a critical condition. Case 5 was a 2-month-old infant without a fever or cough, whose parents reported that the infant displayed drowsiness and had poor feeding. Among the 5 cases, only case 2 had a clear history of exposure to COVID-19, case 5 had a suspected contact history, while the others had no clear exposure history. The onset symptoms of these patients may not have been related to SARS-CoV-2 infection, or, the symptoms of SARS-CoV-2 infection were relatively hidden or mild before COVID-19 was confirmed. It is also possible that the initial medical and contact history provided by the parents was insufficient for an accurate determination of COVID-19 diagnosis. However, all the cases had pneumonia confirmed by chest CT scan before or soon after admission. Because the pneumonia could not be explained by their primary disease and given that COVID-19 was breaking out in Wuhan, the infants were immediately isolated and tested for SARS-CoV-2 by nucleic acid RT-PCR. The five patients were all infected with the virus.

Most children infected with SARS-CoV-2 show mild clinical manifestations and recover within 1–2 weeks after disease onset (6). Huang et al. reported that 40 (98%) out of 41 adult COVID-19 patients had fever (1). In our report, four (80%) of the five patients had fever during the course of the disease. Considering that these children had coexisting conditions, it was unclear whether their fever was related to the coexisting conditions or the SARS-CoV-2 infection. The first confirmed case of an asymptomatic child with COVID-19 was reported in Shenzhen, China, on 20 January 2020 (7). Our case series is the first report to describe the clinical features of COVID-19 with non-respiratory symptoms as the first manifestation in children, including two severe cases.

Seven coronaviruses are known to cause disease in humans. Of these, HCoV-229E, HCoV-OC43, HCoV-NL63, and HCoV-HKU1 usually cause mild upper respiratory symptoms, and rarely cause serious disease. SARS-CoV and MERS-CoV infections can lead to severe lower respiratory tract symptoms with acute severe respiratory syndrome. SARS-CoV-2 belongs to the betacoronaviruses and can also infect the lower respiratory tract and cause pneumonia. The mortality rate associated with COVID-19 is generally lower than that of SARS and MERS (5, 8–10).

Similar to adults (1, 6, 10), in the early phase of COVID-19 infection in our patients, the white blood cell count was normal or decreased, while the absolute number of lymphocytes was reduced. CRP levels were normal or slightly elevated some of the time, and those of PCT were normal in three of our patients (cases 2, 3, and 5). In severe cases (cases 1 and 4), CRP and PCT levels were significantly increased, while the levels of liver enzymes, muscle enzymes, and myohemoglobin were increased. Additionally, coagulation was abnormal, and the D-dimer level was increased in case 1. In the early phase of the disease, chest X-rays showed no abnormal changes, thereby facilitating misdiagnosis. Chest CT examinations showed the presence of viral pneumonia, with early lesions often located in the lung periphery and showing ground-glass opacity and/or infiltrating shadows. With disease progression, the lesions increased, expanded, and involved multiple lung lobes. Some of the lesions consolidated and coexisted with ground-glass opacities or strip shadows. In the serious cases, the CT images showed diffuse consolidation in the lungs, and some showed “white lung” and air bronchogram (11–13).

Importantly, four of the five cases had digestive tract symptoms as the first manifestation. Wang et al. reported that 10.1% of adult COVID-19 patients initially presented with diarrhea and nausea 1 to 2 days before the development of fever and dyspnea (10). The reason for this phenomenon might be related to the distribution of receptors and the transmission pathway associated with SARS-CoV-2 infection in the host. SARS-CoV-2, like SARS-CoV, infects the host *via* the receptor ACE2 (14, 15). ACE2 is abundantly expressed in alveolar type I and type II epithelial cells and small intestinal epithelial cells. It is also present in arterial and venous endothelial cells and arterial smooth muscle cells of all organs (16, 17). This suggests that SARS-CoV-2 might infect patients not only through the respiratory tract in the form of air droplets, but also through the digestive tract, by contact or fecal–oral transmission. Children are active and do not pay much attention to hand hygiene. Consequently, the likelihood of infection through the digestive tract by contact or fecal–oral transmission is likely to be significantly greater for children than for adults, and gastrointestinal symptoms as the first manifestation might be more common in children. Backer et al. estimated that the mean incubation period for COVID-19 was 6.4 days (range: 2.1–11.1 days, 2.5th to 97.5th percentile), with potential asymptomatic transmission (18, 19). This might explain why case 3, who was admitted with head trauma but with no respiratory symptoms, tested positive for SARS-CoV-2 nucleic acid and his lung CT scan showed pneumonia. Rodent pulmonary ACE2 expression is developmentally regulated. ACE2 mRNA levels were observed to peak at E14 and were lowest when mice reached adulthood, whereas the protein abundances showed the opposite trend (17). It remains to be clarified whether there is a similar ACE2 expression pattern in humans. If the expression of human ACE2 is also developmentally regulated, it might explain why the incidence and severity of COVID-19 are higher in adults than in children.

Similar to that observed for SARS and MERS, children infected with SARS-CoV-2 exhibit relatively mild symptoms and the incidence of infection is lower than in adults. Although the specific reasons are not clear, it has been suggested that this might be related to an immature immune system in children (20, 21). Nevertheless, there were still two severe cases of COVID-19 among these five patients. These two patients had multiple organ dysfunction (liver, kidney, and myocardium), significantly increased plasma levels of IL-6 and IL-10, and abnormal cellular immune function (decreased counts of CD3+ and CD4+ T lymphocytes and CD16 + CD56 NK cells), suggesting that in addition to the direct damage to organs caused by the virus, further damage to organ function occurred that was related to the cytokine storm induced by viral invasion (1, 10).

Studies on adults have shown that severe cases of COVID-19 are often accompanied by underlying conditions (hypertension, diabetes, cardiovascular disease, cerebrovascular disease) (1, 10). Among the five patients in our report, two were critically ill and presented with underlying or coexisting conditions (case 1, intussusceptions; case 4, rotavirus infection, hydronephrosis, and a stone in the left kidney). However, it is unclear why the other two patients did not become critically ill, even though they also had underlying or coexisting conditions (case 2, acute suppurative appendicitis perforation with localized peritonitis; case 3, right frontal subdural hemorrhage with convulsion caused by brain injury). The indisputable fact is that during the outbreak of COVID-19, pediatric emergency departments are experiencing a significant reduction in visits. One of the important reasons may be that parents, afraid of being infected with SARS-CoV-2, do not bring ill children to the hospital in time, delaying treatment of their children, which then leads to the occurrence of serious complications. Whether this can explain the two critical patients (case 1 and case 4) in this report needs to be further explored. Nevertheless, the different manifestations of COVID-19 in young children raise intriguing questions regarding the pathophysiology and spread of this disease. In particular, the role of the host's immune response to the causative agent in the disease process, requires further investigation.

The outbreak of SARS-CoV-2 is a major challenge for clinicians. The pathogenesis of COVID-19 remains to be elucidated and there is no effective antiviral treatment to date (22). Some antiviral drugs such as lopinavir/ritonavir, arbidol, interferon alpha-2b, and some traditional Chinese medicines were tried in adult patients (1, 23). Considering the side effects of these drugs and based on the recommendations of the Pediatric Society of the Chinese Medical Association (6), we chose only interferon alpha-2b and ribavirin for antiviral treatment. Two critically ill children (cases 1 and 4) had a severe systemic inflammatory reaction. CT scans revealed progression of the lesion. They were being treated with mechanical ventilation and blood purification (plasma exchange, CRRT). Although there is controversy regarding whether glucocorticoids should be used for critical cases of COVID-19 (6), we nonetheless used methylprednisolone [2 mg/(kg·day)] for a short period (3–5 days).

Our study had some limitations. First, all five cases were confirmed by detection of SARS-CoV-2 nucleic acid in throat swab samples, and false-positive results can occur. We did not test other samples of the patients (such as stool and blood). However, through a combination of exposure history, clinical manifestations, and chest CT scan results we could were reasonably certain that these five cases were confirmed with SARS-CoV-2 infection. Second, owing to the sudden outbreak of COVID-19, SARS-CoV-2 detection kits were in short supply, and some of the patients could not be immediately tested. The sensitivity and specificity of the kits can also differ, which can lead to false-positive or false-negative results. We are certain that four of the five patients in this case series had SARS-CoV-2 infection before admission. Patient 4 was tested three times for SARS-CoV-2 after admission, and only tested positive the third time and it was unclear whether SARS-CoV-2 infection occurred before or after admission. However, he had fever and shortness of breath for half a day in the emergency observation room. Although he had no other respiratory symptoms, we immediately decided to perform a chest CT scan before admission and found some consolidation in the images (Figures 4A,B). This implied that SARS-CoV-2 infection occurred before admission. Third, we only analyzed five cases of COVID-19 in children showing non-respiratory symptoms as the first manifestation when they were admitted from the emergency department. The true incidence of similar cases requires further study.

Based on our experience, in regions with significant COVID-19 incidence, we suggest consideration of an approach similar to that in our hospital, with SARS-CoV-2 nucleic acid testing and chest CT scans carried out for children with COVID-19 exposure history and/or fever. All suspected and confirmed patients should be admitted to isolation wards. If there is no fever or exposure history, a pediatric patient with gastrointestinal symptoms may not need to be screened for COVID-19, particularly if testing kits are limited.

In brief, COVID-19 in children is relatively occult or mild, and it is easy to miss the diagnosis in the early stages when present with a non-respiratory disease. Severe COVID-19 can also occur in children with underlying or coexisting diseases. In epidemic areas, the possibility of SARS-CoV-2 infection should be suspected when children show digestive tract symptoms, especially with fever and/or exposure history.

## Data Availability Statement

The original contributions presented in the study are included in the article/supplementary material, further inquiries can be directed to the corresponding author/s.

## Ethics Statement

The studies involving human participants were reviewed and approved by Institutional ethics board of Wuhan children's Hospital Affiliated to Tongji Medical College of Huazhong University of science and technology. Written informed consent to participate in this study was provided by the participants' legal guardian/next of kin. Written informed consent was obtained from the individual(s), and minor(s)' legal guardian/next of kin, for the publication of any potentially identifiable images or data included in this article.

## Author Contributions

XC designed the study, drafted the initial manuscript, reviewed, and revised the manuscript. YM, SL, YC, and ZR designed the data collection instruments, collected data, and reviewed and revised the manuscript. WL designed the study, coordinated and supervised data collection, and critically reviewed the manuscript for important intellectual content. All authors approved the final manuscript as submitted and agree to be accountable for all aspects of the work.

## Conflict of Interest

The authors declare that the research was conducted in the absence of any commercial or financial relationships that could be construed as a potential conflict of interest.

## Footnotes

^1^https://www.who.int/emergencies/diseases/novel-coronavirus-2019/situation-reports/

## Abbreviations

COVID-19: coronavirus disease 2019
SARS-CoV-2: severe acute respiratory syndrome coronavirus 2
WHO: World Health Organization
ICTV: the International Committee on Taxonomy of Viruses
MERS-CoV: Middle East respiratory syndrome coronavirus
CT: computed tomography
PICU: pediatric intensive care unit
NK: natural killer
CRRT: continuous renal replacement therapy
CRP: C-reactive protein
PCT: procalcitonin
PT: prothrombin time
APTT: activated partial thromboplastin time
IL: interleukin
EEG: electroencephalogram
ACE2: angiotensin converting enzyme 2.
